# Incidence of Diabetes Mellitus and Its Impact on Outcomes in Patients Undergoing Surgical Pancreatectomy for Non-Malignant and Malignant Pancreatobiliary Diseases—A Retrospective Analysis

**DOI:** 10.3390/jcm12247532

**Published:** 2023-12-06

**Authors:** Anna Schranz, Christoph Sternad, Faisal Aziz, Doris Wagner, Peter Kornprat, Robert Sucher, Philipp J. Jost, Albert Wölfler, Thomas R. Pieber, Harald Sourij, Jakob M. Riedl, Felix Aberer

**Affiliations:** 1 Department of Internal Medicine, Division of Endocrinology and Diabetology, Medical University of Graz, 8036 Graz, Austria; anna.schranz@stud.medunigraz.at (A.S.); christoph.sternad@stud.medunigraz.at (C.S.); faisal.aziz@medunigraz.at (F.A.); thomas.pieber@medunigraz.at (T.R.P.); felix.aberer@medunigraz.at (F.A.); 2 Department of Surgery, Medical University of Graz, 8036 Graz, Austria; doris.wagner@medunigraz.at (D.W.); peter.kornprat@medunigraz.at (P.K.); robert.sucher@medunigraz.at (R.S.); 3 Department of Internal Medicine, Division of Oncology, Medical University of Graz, 8036 Graz, Austria; philipp.jost@medunigraz.at (P.J.J.); j.riedl@medunigraz.at (J.M.R.); 4 Department of Internal Medicine, Division of Hematology, Medical University of Graz, 8036 Graz, Austria; albert.woelfler@medunigraz.at

**Keywords:** pancreatic cancer, pancreoprivic diabetes, pancreatobiliary surgery, diabetes type 3c, mortality

## Abstract

Diabetes mellitus (DM) is a prominent risk factor for malignant and non-malignant pancreatic diseases. Furthermore, the presence of DM predicts an unfavourable outcome in people with pancreatic cancer. This retrospective observational study investigated 370 patients who underwent pancreatic resection surgery for various indications (84.3% in malignant indication) in a single surgery centre in Graz, Austria. The preoperative and postoperative diabetes statuses were evaluated according to surgery method and disease entity and predictors for diabetes development after surgery, as well as outcomes (survival and cancer recurrence) according to diabetes status, were analysed. In the entire cohort, the postoperative diabetes (postopDM) incidence was 29%. PostopDM occurred significantly more frequently in malignoma patients than in those with benign diseases (31.3% vs. 16.7%; *p* = 0.040, OR = 2.28). In the malignoma population, BMI, longer surgery duration, and prolonged ICU and hospital stay were significant predictors of diabetes development. The 1- and 2-year follow-ups showed a significantly increased mortality of people with postopDM in comparison to people without diabetes (HR 1-year = 2.02, *p* = 0.014 and HR 2-years = 1.56, *p* = 0.034). Local cancer recurrence was not influenced by the diabetes status. Postoperative new-onset diabetes seems to be associated with higher mortality of patients with pancreatic malignoma undergoing pancreatobiliary surgery.

## 1. Introduction

Pancreatic carcinoma ranks fourth among the most common malignancy-related causes of death in Europe with a 5-year survival rate of approximately 5% [1,2]. In 2021, 84,200 deaths due to pancreatic carcinomas were reported in the European Union [2]. Only 15–20% of pancreatic cancer patients qualify for curative therapy through pancreatic resection. However, the curative approach is still associated with a very poor prognosis, with only 20% of affected people being alive 5 years after surgery [1].

Diabetes, mainly type 2, is a well-known risk factor for numerous malignant diseases, particularly for ductal adenocarcinomas of the pancreas [3,4,5]. Also, diabetes occurring in response to chronic pancreatitis is associated with an increased risk for pancreatic malignancy [6,7].

A meta-analysis revealed that diabetes (RR: 1.4–2.2), along with tobacco use (RR: 1.5–2.2), chronic pancreatitis (RR: 2.7–5.1), alcohol consumption (RR: 1.1–1.5), obesity (RR: 1.2–1.5), and a positive family history (RR: 1.7–1.8), are major risk factors for pancreatic carcinoma [5].

Insulin resistance, beta cell dysfunction, hyperglycaemia, chronic inflammation, and oxidative stress, being present in type 2 diabetes, have been suggested as underlying mechanisms promoting the risk of pancreatic cancer [3,4].

At the time of diagnosis, approximately 85% of individuals with pancreatic carcinoma already have impaired glucose tolerance [8]. An estimated 50% of patients with pancreatic carcinoma have diabetes at diagnosis, with diabetes being diagnosed 2–3 years prior to cancer diagnosis in 50–75% of cases [4,8,9].

New-onset diabetes seems to have a higher impact on pancreatic cancer risk than previously established diabetes. An explanation could be that new-onset diabetes can be an early, unfortunately often overlooked, symptom of pancreatic carcinoma rather than a potentially causal risk factor [8,10]. In a cohort study from the USA, 1% of individuals (>50 years) with new-onset diabetes were diagnosed with pancreatic carcinoma within 3 years of diabetes diagnosis [11]. Therefore, a pancreatic origin of diabetes should be considered and a generous indication for further diagnostic tests (e.g., ultrasound or CT) seems reasonable in atypical cases.

Controversial data exist regarding the relationship between diabetes and the outcome of patients with pancreatic carcinomas. Some studies indicate that diabetes is associated with increased mortality in patients with resected pancreatic carcinomas [12,13,14,15], while others suggest no association [16]. The relationship between diabetes duration and mortality also remains unclear. A meta-analysis of 17 observational studies between 1996 and 2016 showed significantly higher mortality in new-onset diabetes (duration < 2 years before carcinoma diagnosis) but not in individuals with previous long-term diabetes [15]. Other studies contradict this finding and identify long-term and new-onset diabetes [12], or just long-term diabetes, as independent risk factors for mortality [14,17]. Similarly, a study from the USA found significantly increased mortality of pancreatic carcinoma patients with diabetes compared to those without diabetes, but not in the group of pancreatic resected individuals [18].

Patients with diabetes have been found to have more advanced tumour stages and larger neoplasms at the time of diagnosis compared to those without diabetes [12,13,16]. Additionally, new-onset diabetes (duration < 2 years before carcinoma diagnosis) could be associated with more aggressive tumour behaviour, as it has been linked to increased mortality and shorter disease-free survival [19].

Data regarding the impact of postoperative new-onset diabetes on the prognosis of patients with pancreatic malignoma are limited. A prospective study published in 2016 found no significant difference between individuals with and without postoperative diabetes. However, in contrast to our study, the cohort was smaller and only ductal adenocarcinomas of the pancreas were considered [20]. This retrospective observational study aimed to evaluate the risk of diabetes in a cohort of pancreas-resected patients for both benign and malignant indications and to further characterize the impact of diabetes on mortality in people with malignant disease.

## 2. Materials and Methods

### 2.1. Study Design and Data Recruitment

This was a single-centre retrospective analysis, which was reported to and approved by the Ethics Committee of the Medical University of Graz (34-051 ex 21/22 dated 14 September 2021). The selection of patients was conducted using the surgery registry of the Division of General Surgery at the University Hospital of Graz, Austria. Patients with pancreatic resection between 2016 and 2022 were selected as the study population. The medical documentation system “MEDOCS” and the medical procedure catalogue for 2022 provided by the Federal Ministry of Social Affairs, Health, Care, and Consumer Protection (BMSGPK) were used for patient identification. The keywords “pancreatic resection” and “pancreatectomy” were defined as relevant search terms corresponding to codes HN050–HN110 in the medical procedure catalogue. These codes were utilized in the “MEDOCS search” to collect the names and identification numbers of eligible patients. The necessary medical data of the selected patients were extracted in collaboration with the Institute for Medical Informatics (IMI) from the medical documentation system “MEDOCS”.

### 2.2. Selection of the Study Population

We included adult patients with a history of partial or total pancreatectomy in response to (suspected) malignant pancreatic neoplasm or benign pancreatic lesions (pseudocysts or necrosis in chronic pancreatitis) or benign solid tumours. Patients with traumatic pancreatic injury, pancreatic resection due to underlying splenic disease (tumour, abscess), pancreatic resection for metastasis removal from an extrapancreatic malignant neoplasm, or patients receiving pancreas transplantation were not considered in the analysis.

### 2.3. Parameters and Surgery-Specific Information of Interest

#### 2.3.1. Baseline Characteristics

The preoperative status, including sex, age, body mass index (BMI), comorbidities (arterial hypertension, atrial fibrillation, coronary heart disease), diabetes status, Charlson Comorbidity Index (CCI), kidney function (serum creatinine and estimated glomerular filtration rate), HbA1c (if available), and the American Society of Anesthesiologists (ASA) score were obtained.

#### 2.3.2. Peri- and Postoperative Assessments

The specific surgical procedure was considered according to the medical procedure catalogue of the BMSGPK 2022 within HN50–HN110. The duration of the surgery, defined as the time (in minutes) between the incision and wound closure time, was assessed. Histological findings, including TNM classification and tumour entity, were recorded and used as criteria for determining whether the tumour was benign or malignant.

The number of days spent in the intensive care unit and the general ward were evaluated.

#### 2.3.3. Postoperative Blood Sugar Level, Diabetes Status, and Diabetes Therapy at Discharge

The maximum and mean values of the measured blood sugar levels were determined for the first and second postoperative week. The postoperative diabetes status was categorized into three groups:

- noDM: neither preoperative nor postoperative (up to day 13 after surgery or the day of hospital discharge) diabetes fulfilling diagnostic criteria (no diabetes diagnosis or glucose lowering therapy at discharge);
- postopDM: postoperative new-onset diabetes (initiation of blood-sugar-lowering therapy at discharge) fulfilling diagnostic criteria;
- preopDM: diabetes being present already prior to surgery.

For individuals with preopDM or postopDM, oral diabetes medication and insulin therapy (drug and injection regimen) at the time of hospital discharge were documented.

#### 2.3.4. Local Cancer Recurrence and Death

If the event of local cancer recurrence occurred, the date of diagnosis was recorded and the time (in months) since surgery was calculated. In the cases where the patient died, the date of death, the cause of death (death due to a malignant primary disease or other causes), and the survival time (in months) beginning with the surgery were documented.

### 2.4. Primary Objective

The primary endpoint was overall survival/time-to-mortality (from time of surgery on) in individuals with pancreatic malignoma.

### 2.5. Secondary Objectives

- Time to local cancer recurrence.
- The incidence of postoperative new-onset diabetes after pancreatic surgeries in the total cohort (benign and malignant).

### 2.6. Statistical Analyses

IBM SPSS Statistics Version 26 was used for statistical analysis. Due to the heterogeneity of patients included, the population was divided into three groups of interest:

- Total population: Individuals with malignant or benign pancreatic neoplasms;
- Population A: Individuals with malignant pancreatic neoplasms;
- Population B: Individuals with benign pancreatic neoplasms or other benign pancreatic diseases.

For descriptive analyses, chi-square or Mann–Whitney U tests were used. Analyses regarding survival and cancer recurrence were performed for Population A using Kaplan–Meier curves, log-rank tests, and Cox regression models. For survival analyses and assessing cancer recurrence, Population A was divided into four groups based on postoperative diabetes status:

- Group 1: noDM;
- Group 2: postopDM;
- Group 3: preopDM;
- Group 4: Combined patients with preopDM or postopDM.

## 3. Results

### 3.1. Baseline Characteristics

Of the 370 identified patients, 84.3% (N = 312) had a malignant and 15.7% (N = 58) a benign disease requiring pancreatic surgery. Further baseline characteristics of the total cohort are given in Table 1.

### 3.2. Primary Endpoint

#### Mortality in Patients with Cancer (Population A) (Figure 1 and Figure 2)

Of the 312 patients who underwent surgery for malignant indication, 63 (20.2%) had a diabetes diagnosis prior to surgery. A total of 78 (31.3%) of the 249 patients without preoperative diabetes fulfilled diabetes diagnosis criteria after surgery and 171 (68.7%) remained without diabetes diagnosis until discharge.

After one, two, and three years of observation 68 (22.7%), 116 (45.1%), and 127 (53.4%), respectively, of all cancer patients had died.

In the first year, the presence of a preopDM or postopDM was significantly associated with mortality when compared to noDM (HR 1.42 and 1.82, respectively). After two years, postopDM, but not preopDM, had a significantly higher mortality when compared to noDM (HR 1.576 and 0.929). After three years, no significant results were seen when comparing the three groups. Table 2 indicates the log-rank test according to the specific diabetes status. The Cox regression analyses demonstrate the hazard ratios for mortality after adjustment for sex, arterial hypertension, atrial fibrillation, coronary artery disease, age, BMI, creatinine, GFR, ASA score, and 10-year-survival based on CCI (Table 3).

**Figure 1:**
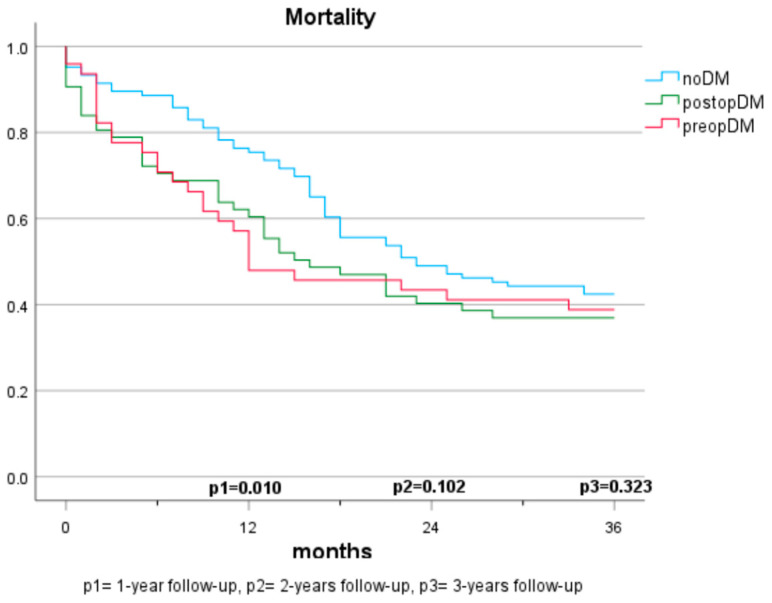
Mortality based on diabetes status (3 groups).

**Figure 2:**
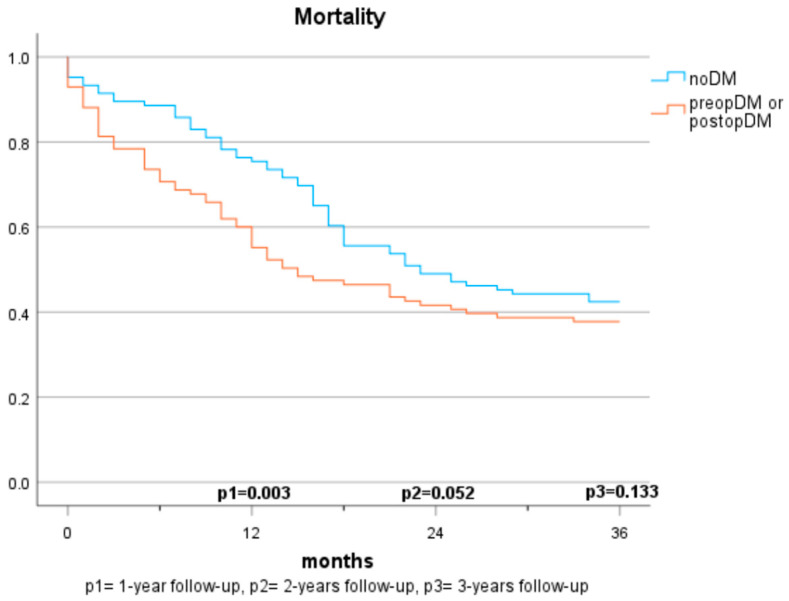
Mortality based on diabetes status (2 groups).

### 3.3. Secondary Endpoints

#### 3.3.1. Local Cancer Recurrence

Cancer recurrence was defined as diagnosis of local pancreatic cancer recurrence. After one, two, and three years of observation 21 (7%), 31 (12.1%), and 32 (13.4%) of all cancer patients, respectively, had been diagnosed with local cancer recurrence.

No significant differences were observed between the diabetes groups. The Cox regression analyses demonstrate the hazard ratios for local cancer recurrence after adjustment for sex, arterial hypertension, atrial fibrillation, coronary artery disease, age, BMI, creatinine, GFR, ASA score, and 10-year survival based on CCI (Table 4).

#### 3.3.2. Diabetes Incidence

Approximately 29% (n = 86) of the study participants who did not have diabetes preoperatively (n = 297) were diagnosed with new-onset diabetes after surgery (defined as the initiation of antidiabetic therapy at discharge). The postoperative diabetes incidence was significantly higher in people with malignant neoplasms, being 31.3% (n = 78) compared to people with benign diseases at 16.7% (n = 8) (*p* = 0.040, OR = 2.28). There was no significant difference by gender (females: 26.9%, n = 43, males: 31.4%, n = 43, *p* = 0.393).

Regarding surgical procedures, new-onset diabetes was observed in 9% (n = 20) of cases following partial pancreatectomy, and, surprisingly, only in 86.8% (n = 66) following total pancreatectomy (*p* < 0.001, OR = 66.33) (Table 5).

Patients with malignant pancreatic diseases and postopDM had a significantly higher BMI compared to the noDM group (25.9 ± 3.9 vs. 24.6 ± 3.9 kg/m^2^, *p* = 0.029).

The duration of surgery and the length of stay in the intensive care unit or general ward varied significantly among individuals with pancreatic malignancies in the different diabetes groups (Table 6).

#### 3.3.3. Therapy for Type 3c Diabetes

For the majority of patients with postopDM (n = 86), insulin therapy was initiated using the basal-bolus regime, employing a combination of long-acting and short-acting insulin preparations (n = 70, 81.4%). Four individuals (4.7%) received therapy just using short-acting insulin. Eight patients (9.3%) were managed with long-acting insulin preparations without short-acting insulin. Additionally, four individuals (4.7%) were prescribed premixed insulin therapy.

## 4. Discussion

### 4.1. Mortality

The mortality rates among patients with pancreatic carcinomas vary widely in the literature and are strongly influenced by cancer stage. In Austria, a 1-year mortality rate of 61% (2017–2018) and a 5-year mortality rate of 89% were reported previously for patients diagnosed with pancreatic cancer [21]. In this study, the 1-year mortality rate was considerably lower at 22.7%. This discrepancy can be explained by considering that only cases with resectable carcinoma were included, which typically have lower tumour stages and a curative treatment approach. Regarding the impact of pre-existing diabetes on survival, there are no consistent findings. In some studies, preopDM affects the mortality negatively [12,13,22,23,24], while other publications do not show significant differences [16,17,20,25]. The results of our study demonstrate a significantly shortened survival in the 1-year follow-up compared to individuals without diabetes. However, in the 2- and 3-year follow-ups and multivariate analyses, the significance is lost. The influence of postoperative new-onset diabetes on mortality has been sparsely studied. Compared to Balzano et al. [20], our study suggests that postoperative new-onset diabetes impacts mortality and survival within the first two years. No significant differences are observed over three years, but a similar trend is discernible. The lack of significance may be due to the smaller sample size in the 3-year follow-up, but it may also be attributed to the generally high mortality in this depicted cohort. While postoperative DM was identified as a significant predictor for mortality in our cohort, no statistical difference between those who had diabetes prior to surgery and those who developed diabetes after surgery was observed. It is supported by evidence that patients with diabetes face a higher risk for acute postoperative complications, such as infection, renal failure, as well as cardiovascular complications [26]. This potentially reflects our findings that mortality risk was specifically increased in the first year after surgery in both populations with diabetes (preopDM and postopDM), but not when year 2 and year 3 after surgery were considered. The fact that glycaemia might play an important role as a biomarker of general health status leaves open the question of whether people with diabetes were generally sicker than people without diabetes. Hence, the question of whether glucose and the quality of glycaemic control represent modifiable risk factors for unfavourable outcomes remains unanswered by our study. Prospective intervention studies in adequately powered cohorts are needed to find this out.

### 4.2. Local Cancer Recurrence

The analyses did not reveal significant differences in terms of local recurrences risks based on diabetes status. This could possibly be due to the higher and earlier mortality of people with diabetes (preoperative and postoperative) compared to individuals without diabetes. Balzano et al. [20] published similar results, indicating no significantly increased risk of local recurrences in people with long-standing or postoperative diabetes. Overall, the evidence regarding the influence of preoperative diabetes mellitus is not clear—there are indications that preoperative diabetes does not increase the risk of recurrence [19,20], or that, on the contrary, it has an impact on cancer recurrence risk [16,27]. It must be recognised as a limitation that only local cancer recurrence and not general cancer relapse was investigated.

### 4.3. Diabetes Incidence

This study revealed that diabetes post-surgery occurred in 29% of the total cohort, in 31.3% of cancer patients, and in 16.7% of non-malignant disorder patients. The overall diabetes incidence of 9% after partial pancreatic resection is most comparable to the previously published data by Lee et al. (13.3% after 30 days) [28]. Our study yielded a tendency towards a lower rate of postoperative diabetes following pancreatic partial resection (ranging from 4.5% to 13.2%, depending on the surgical technique) compared to other studies (13.3% to 43%) [27,28,29,30]. However, since we analysed the diabetes status during the index hospital stay only, our results might underestimate the overall post-surgery incidence. In patients with total pancreatectomy, a postoperative diabetes incidence of 86.8% was observed in our study, suggesting that, by definition, total pancreatectomy may have been planned but pancreatic tissue remained in situ.

As previously demonstrated, also in our study, age was found not to influence postoperative diabetes incidence and postoperative diabetes was more common with higher BMI in patients with pancreatic malignomas [30]. Prolonged surgery and extended stays in intensive care units or regular wards were associated with an increase in the risk of diabetes onset, potentially reflecting longer surgery duration in total pancreatectomies.

### 4.4. Limitations

Several limitations arise from the retrospective design of the study. Firstly, it should be mentioned that documentation gaps, especially regarding preoperative and postoperative diabetes, cannot be ruled out. For instance, a preoperative HbA1c was available in only 112 individuals, which might mean that people were wrongly classified as they might have had diabetes already prior to surgery. In contrast to other publications, the classification of preoperative diabetes status was not further analysed with regard to different diabetes durations given the limited sample size. Moreover, we were not able to collect in retrospect standardised glucose readings that would allow proper analyses of glycaemic control and mortality outcomes. We also acknowledge, that given the higher rate of post-surgery diabetes in those having total pancreatectomy, our data might be biased as the underlying tumour stage might differ between the groups, potentially impacting survival. In addition, we must consider as a limitation the single-centre design of the study, which might not allow transfer to the global population of patients who underwent pancreatic surgery. Furthermore, our study cannot rule out that after the index hospital stay additional people developed diabetes.

## 5. Conclusions

Postoperative new-onset diabetes mellitus seems to be associated with the outcome of patients with pancreatic malignomas. The limited available literature on this topic underlines the need for prospective studies with larger sample sizes to make definitive and comparable statements. Furthermore, a subject of future research remains to evaluate whether peri- and postoperative blood glucose control has an impact on the long-term outcome of these patients.

## Figures and Tables

**Table 1:** Baseline characteristics. *p* indicates statistical significance.

		Total Population n = 370	Population A n = 312	Population B n = 58	*p*
Females ^1^		187 (50.5)	156 (50)	31 (53.4)	0.630
Arterial hypertension ^1^		200 (54.1)	172 (55.1)	28 (48.3)	0.336
Atrial fibrillation ^1^		31 (8.4)	29 (9.3)	2 (3.4)	0.140
Coronary artery disease ^1^		48 (13.0)	44 (14.1)	4 (6.9)	0.134
Diabetes status preoperative ^1^	noDM	297 (80.3)	249 (79.8)	48 (82.2)	0.700
	DM 1	3 (0.8)	3 (1)	0 (0)	
	DM 2	70 (18.9)	60 (19.2)	10 (17.2)	
Age ^1^		69 ± 11	70 ± 11	63 ± 12	**<0.001**
BMI ^1^		25.1 ± 4.3	25.0 ± 4.0	25.6 ± 5.6	0.609
Creatinine (mg/dL) ^2^		0.87 ± 0.27	0.87 ± 0.24	0.84 ± 0.41	0.259
eGFR (mL/min/1.73 m^2^) ^2^		81.53 ± 18.16	81.53 ± 17.16	81.60 ± 22.66	0.124
HbA1c preoperative (mmol/mol) ^2,3^		40.0 ± 13.5	40.0 ± 13.6	38.5 ± 12.6	0.430
ASA score ^2^		2 ± 0.7	2 ± 0.7	2.5 ± 0.7	0.853
CCI score ^2^		7 ± 2.7	7 ± 1.9	2 ± 1.8	**<0.001**
Partial pancreatectomy ^1^		268 (72.4)	217 (69.6)	51 (87.9)	
Total pancreatectomy ^1^		102 (27.6)	95 (30.4)	7 (12.1)	
Pancreatectomy left ^1^		93 (25.1)	67 (21.5)	26 (44.8)	**<0.001**
Partial pancreatectomy with preservation of the pylorus ^1^		75 (20.3)	65 (20.8)	10 (17.2)	0.532
Pancreatectomy right with preservation of the duodenum ^1^		1 (0.3)	0 (0)	1 (1.7)	
Whipple ^1^		99 (26.8)	85 (27.2)	14 (24.1)	0.624
Total pancreatectomy ^1^		56 (15.1)	52 (16.7)	4 (6.9)	
Extended total pancreatectomy ^1^		46 (12.4)	43 (13.8)	3 (5.2)	

^1^ n (%), ^2^ Median and SD, ^3^ HbA1c data are missing of: 258 (total population), 218 (population A), and 40 (population B) people, BMI (body mass index), eGFR (estimated glomerular filtration rate), ASA score (American Society of Anesthesiologists score), CCI (Charlson Comorbidity Index).

**Table 2:** Mortality, Log-rank Test.

		NoDM	PostopDM	PreopDM	PreopDM or PostopDM	*p*_a_	*p*_b_	*p*_c_	*p*_d_
1-year		n = 164	n = 76	n = 60	n = 136				
	mortality ^1^	26(15.9)	23(30.3)	19(31.7)	42(30.9)	0.863	**0.009**	**0.012**	**0.003**
2-years		n = 137	n = 65	n = 55	n = 120				
	mortality ^1^	55(40.1)	36(55.4)	25(45.5)	10(50.8)	0.429	0.307	**0.032**	0.052
3-years		n = 125	n = 64	n = 49	n = 113				
	mortality ^1^	62(49.6)	38(59.4)	27(55.1)	65(57.5)	0.918	0.264	0.176	0.133

^1^ n (%), *p*_a_ = comparison of preopDM and postopDM, *p*_b_ = comparison of preopDM and noDM, *p*_c_ = comparison of postopDM and noDM, *p*_d_ = comparison of noDM and preopDM or postopDM.

**Table 3:** Mortality, Cox regression.

		Unadjusted HR	95%CI	*p*	Adjusted HR ^1^	95%CI ^1^	*p* ^1^
1-year	noDM	1			1		
	postopDM	2.019	1.152–3.538	**0.014**	1.821	1.024–3.239	**0.041**
	preopDM	2.136	1.182–3.860	**0.012**	1.427	0.733–2.777	0.295
2-years	noDM	1			1		
	postopDM	1.560	1.024–2.375	**0.034**	1.576	1.022–2.431	**0.040**
	preopDM	1.269	0.791–2.037	0.324	0.929	0.546–1.580	0.786
3-years	noDM	1			1		
	postopDM	1.311	0.875–1.964	0.189	1.366	0.899–2.075	0.144
	preopDM	1.283	0.816–2.017	0.281	1.020	0.608–1.711	0.941

^1^ adjusted for sex, arterial hypertension, atrial fibrillation, coronary artery disease, age, body mass index, creatinine, glomerular filtration rate, American Society of Anesthesiologists score, 10-year survival based on Charlson Comorbidity Index.

**Table 4:** Risk of local cancer recurrence, Cox regression.

		HR	CI	*p*	HR ^1^	CI ^1^	*p* ^1^
1-year	noDM	1			1		
	postopDM	1.078	0.375–3.105	0.889	1.031	0.349–3.050	0.956
	preopDM	1.406	0.488–4.048	0.528	1.136	0.350–3.685	0.832
2-years	noDM	1			1		
	postopDM	0.806	0.320–2.031	0.647	0.823	0.314–2.154	0.691
	preopDM	1.171	0.489–2.087	0.723	0.993	0.353–2.790	0.989
3-years	noDM	1			1		
	postopDM	1.023	0.463–2.263	0.954	1.034	0.449–2.380	0.937
	preopDM	0.637	0.217–1.874	0.413	0.428	0.125–1.472	0.178

^1^ adjusted for sex, arterial hypertension, atrial fibrillation, coronary artery disease, age, BMI, creatinine, glomerular filtration rate, American Society of Anesthesiologists score, 10-year survival based on Charlson Comorbidity Index.

**Table 5:** PostopDM incidence based on surgery technique.

	PostopDM			
	Total Population n = 297 ^2^	Population n = 249 ^2^	Population B n = 48 ^2^	*p* *
Pancreatectomy left ^1^	7 (9.1) of 77	4 (7.4) of 54	3 (13) of 23	0.431
Partial pancreatectomy with preservation of the pylorus ^1^	3 (4.5) of 67	3 (5.2) of 58	0 (0) of 9	0.421
Whipple ^1^	10 (13.2) of 76	10 (15.2) of 66	0 (0) of 10	0.187
Pancreatectomy right with preservation of the duodenum ^1^	0 (0) of 1	0 (0) of 0	0 (0) of 1	
Total pancreatectomy ^1^	35 (89.7) of 39	32 (88.9) of 36	3 (100) of 3	0.496
Extended total pancreatectomy ^1^	31 (83.8) of 37	29 (82.9) of 35	2 (100) of 2	0.522
Partial pancreatectomy ^1^	20 (9.0) of 223	17 (9.6) of 178	3 (7.0) of 43	0.536
Total pancreatectomy ^1^	66 (86.8) of 76	61 (85.9) of 71	5 (100) of 5	0.345

^1^ n (%), ^2^ only patients with the preoperative diabetes status “noDM” are included, *p* *: comparison population A and B.

**Table 6:** PostopDM incidence based on baseline characteristics.

		Total Population n = 297			Population A n = 249			Population B n = 48		
		PostopDM	NoDM	*p*	PostopDM	NoDM	*p*	PostopDM	NoDM	*p*
Sex ^1^	female	43(26.9)	117(73.1)	0.393	38(28.6)	95(71.4)	0.316	5(18.5)	22(81.5)	0.696
	male	43(31.4)	94(68.6)		40(34.5)	76(65.5)		3(14.3)	18(85.7)	
Arterial hypertension ^1^	yes	46(31.1)	102(68.9)	0.421	42(33.6)	83(66.4)	0.437	4(17.4)	19(82.6)	0.897
	no	40(26.8)	109(73.2)		36(29.0)	88(71.0)		4(16.0)	21(84.0)	
Atrial fibrillation ^1^	yes	6(33.3)	12(66.7)	0.673	5(31.3)	11(68.7)	0.995	1(50.0)	1(50.0)	0.196
	no	80(28.7)	199(71.3)		73(31.3)	160(68.7)		7(15.2)	39(84.8)	
Coronary heart disease ^1^	yes	10(35.7)	18(64.3)	0.407	10(40.0)	15(60.0)	0.324	0(0)	3(100)	0.424
	no	76(28.3)	193(71.7)		68(30.4)	156(69.6)		8(17.8)	37(82.2)	
Age ^2^		70 ± 9.4	67 ± 11.9	0.090	70 ± 9	69 ± 12	0.245	66.5 ± 13	62 ± 12.40	0.674
BMI ^2^		25.7 ± 3.99	24.7 ± 4.21	0.139	25.9 ± 3.9	24.6 ± 3.9	**0.029**	24.6 ± 4.7	25.8 ± 5.3	0.293
Creatinine preoperative [mg/dL] ^2^		0.84 ± 0.24	0.85 ± 0.29	0.580	0.85 ± 0.24	0.85 ± 0.24	0.831	0.80 ± 0.16	0.88 ± 0.47	0.256
eGFR preoperative [mL/min/1.73 m^2^] ^2^		81.27 ± 16.10	82.93 ± 18.61	0.936	81.26 ± 15.58	83.2 ± 17.66	0.761	83.66 ± 20.26	79.75 ± 22.45	0.471
CCI [Punkte] ^2^		7 ± 2.5	6 ± 2.5	0.042	7 ± 1.9	7 ± 1.6	0.297	2 ± 1.2	2 ± 1.7	0.818
Surgery duration [minutes] ^2^		257 ± 87	236 ± 96	**0.008**	271 ± 84	250 ± 92	**0.028**	188 ± 104	163 ± 99	0.438
ICU stay [days] ^2^		3 ± 9	2 ± 3	**<0.001**	3 ± 9	2 ± 3	**<0.001**	2 ± 2	2 ± 2	0.157
Hospital stay [days] ^2^		15 ± 13	12 ± 9	**<0.001**	15 ± 13	12 ± 10	**<0.001**	10 ± 5	10 ± 8	0.488

^1^ n (%), ^2^ Median and SD, BMI (body mass index), eGFR (estimated glomerular filtration rate), CCI (Charlson Comorbidity Index).

## Data Availability

The data presented in this study are available on request from the corresponding author.
